# Validity of a Novel Research-Grade Physical Activity and Sleep Monitor for Continuous Remote Patient Monitoring

**DOI:** 10.3390/s21062034

**Published:** 2021-03-13

**Authors:** Bríd McDevitt, Lisa Moore, Nishat Akhtar, James Connolly, Rónán Doherty, William Scott

**Affiliations:** 1 Department of Computing, Letterkenny Institute of Technology, Letterkenny, F92 FC93 Donegal, Ireland; L00138039@student.lyit.ie; 2 Department of Science, Letterkenny Institute of Technology, Letterkenny, F92 FC93 Donegal, Ireland; L00115999@student.lyit.ie (L.M.); William.Scott@lyit.ie (W.S.); 3 Department of Law & Humanities, Letterkenny Institute of Technology, Letterkenny, F92 FC93 Donegal, Ireland; Ronan.Doherty@lyit.ie

**Keywords:** actigraphy, accelerometer, inertial measurement unit, Digital Healthcare, free-living, supervised protocol, physical activity, activity cut-points, health behaviors, sleep monitoring

## Abstract

In the midst of the COVID-19 pandemic, Remote Patient Monitoring technologies are highly important for clinicians and researchers. These connected-health technologies enable monitoring of patients and facilitate remote clinical trial research while reducing the potential for the spread of the novel coronavirus. There is a growing requirement for monitoring of the full 24 h spectrum of behaviours with a single research-grade sensor. This research describes a free-living and supervised protocol comparison study of the Verisense inertial measurement unit to assess physical activity and sleep parameters and compares it with the Actiwatch 2 actigraph. Fifteen adults (11 males, 23.4 ± 3.4 years and 4 females, 29 ± 12.6 years) wore both monitors for 2 consecutive days and nights in the free-living study while twelve adults (11 males, 23.4 ± 3.4 years and 1 female, 22 ± 0 years) wore both monitors for the duration of a gym-based supervised protocol study. Agreement of physical activity epoch-by-epoch data with activity classification of sedentary, light and moderate-to-vigorous activity and sleep metrics were evaluated using Spearman’s rank-order correlation coefficients and Bland–Altman plots. For all activity, Verisense showed high agreement for both free-living and supervised protocol of r = 0.85 and r = 0.78, respectively. For physical activity classification, Verisense showed high agreement of sedentary activity of r = 0.72 for free-living but low agreement of r = 0.36 for supervised protocol; low agreement of light activity of r = 0.42 for free-living and negligible agreement of r = −0.04 for supervised protocol; and moderate agreement of moderate-to-vigorous activity of r = 0.52 for free-living with low agreement of r = 0.49 for supervised protocol. For sleep metrics, Verisense showed moderate agreement for sleep time and total sleep time of r = 0.66 and 0.54, respectively, but demonstrated high agreement for determination of wake time of r = 0.83. Overall, our results showed moderate-high agreement of Verisense with Actiwatch 2 for assessing epoch-by-epoch physical activity and sleep, but a lack of agreement for activity classifications. Future validation work of Verisense for activity cut-point potentially holds promise for 24 h continuous remote patient monitoring.

## 1. Introduction

In the era of ubiquitous digital connection, Remote Patient Monitoring (RPM) is an expanding and developing area of healthcare research and delivery improvement [1,2,3]. RPM technologies automatically monitor and report on patients’ activity-related vital signs [4,5], oftentimes with chronic conditions [3,6]. In the midst of the COVID-19 pandemic, RPM technologies that enable contactless monitoring of patients are integral for minimising the spread of the novel coronavirus [7,8,9] while accommodating remote clinical research [10,11,12]. 

For human activity monitoring, physical behaviours that occur throughout a full 24 h day are categorised into physical activity (PA), sedentary behaviour (SB) and sleep [13]. These three behaviours are significant for research and health considerations due to their verifiable impact on health [14,15,16], both independently and symbiotically [17,18,19]. Time spent in one behaviour in a 24 h period will directly influence at least one of the other behaviours [19]. Higher sleep quality increases energy and reduces fatigue levels [20,21]. Reciprocally, greater PA ameliorates sleep quality [20,22]. Moreover, the optimal combination between time spent sleeping and in active behaviours (both light and moderate to vigorous physical activities (MVPA)) is associated with lower cardiovascular risk [23]. It is therefore advisable to target all behaviours together [24] in free-living observations of a 24 h day to better comprehend the individual and combined impacts of these activity-related parameters [25]. 

Accelerometer-based monitors that balance cost and feasibility have emerged as valid tools to directly quantify movement [26] that results from PA [27], SB [28,29] and sleep [30,31]. Accelerometers offer low-cost continuous substitution for polysomnography (PSG) [32] and indirect calorimetry [33,34,35], which are the gold standards for sleep and PA monitoring. To date, studies that measured waking movement behaviour and sleep typically utilised two separate accelerometer models [36,37]. Given that waking activity behaviour and sleep can be directly assessed with similar approaches for body movement acceleration detection [38], a logical development for convenience and cost-effectiveness would be to utilise one single accelerometer that can measure PA and sleep over the full 24 h spectrum [22]. Ref. [39] reported the existence of only one recently developed research-specific device that fulfils such requirements: the Actigraph Link (ActiGraph, LLC), therefore advocating for more monitors that can objectively and simultaneously measure waking movement and sleep, and minimise the burden on research cohorts to wear distinct devices that measure behaviours independently [25,40].

The Actiwatch 2 accelerometer (Philips Respironics, Eindhoven, The Netherlands) is a commonly utilised wrist-worn sleep-monitor, that has been validated and widely used for detection of sleep duration and sleep quality [41]. The Actiwatch 2 also facilitates measurement of PA in proprietary activity counts per time unit. A study by [36] developed PA thresholds to segment sedentary, light and MVPA levels of activity for the Actiwatch 2 by comparing activity counts to indirect calorimetry using a portable metabolic cart and an actigraph device [42]. Another study [43] validated the Actiwatch 2 for PA by examination of activity level against energy expenditure measured using indirect calorimetry with the Actiwatch 2. Results were strongly correlated to a widely validated PA device, the ActiGraph wGT3X-BT, thus making the Actiwatch 2 a valid device for both PA and sleep monitoring [36]. This establishes the Actiwatch 2 as a device for the full spectrum of 24 h activity monitoring, which is desirable in clinical research involving participants where participant burden is a pertinent issue for both sleep and PA. This is supported by aforementioned findings based on ActiGraph wGT3X-BT, and other previous studies on its validity in sleep monitoring [43,44] and thus, a suitable device for validation of novel sensors such as that presented in this research. 

The Actiwatch 2 is relatively expensive (approximately US $1500), and produces summative information with a requirement for manual data upload. A viable alternative research-grade accelerometer that can measure waking movement behaviour and sleep, that is more cost-effective, with access to raw sensor data and a long battery life with automated data upload to a secure cloud server, would be beneficial for long-term activity and sleep measurement and assessment. Recently, the use of the accelerometers that provide raw acceleration data in place of a proprietary filtered data units has increased [45,46], with a desired criterion being the production of temporal raw data, as is normally outputted from research-grade monitors [47]. Long-lasting battery life and memory storage is an important consideration to professionals who require high-resolution outputs. However, a necessary equilibrium is the production of detailed data without compromising other practical considerations such as sensor dimensions and burden on participants to wear the device [48]. 

This study implements Verisense, a novel wrist-worn inertial measurement unit (IMU) sensor designed for clinical trials, and developed by Shimmer Research Ltd. (Dublin, Ireland). Verisense accommodates continuous RPM through integration of their wearable sensor, base-station, and cloud platform for automatic data upload. Verisense outputs raw IMU sensor data on waking movement behaviour and sleep and has up to six months of battery life with no recharging. As discussed by [49], these functionalities fit the desirable requirements for sensor systems measuring healthcare parameters in that they uninterruptedly measure and wirelessly report all health-related information after one initial setup, placing minimal restrictions on participants for interaction or maintenance. While Verisense accommodates these requirements, Actiwatch 2 does not, demonstrating a need for Verisense to be validated for future studies. Other alternatives were considered; however, any sensors of similar specifications were either more expensive or lacked in at least one key feature that Verisense offered [50,51]. Furthermore, sensors that differed in body placement location such as shoe-worn devices were deemed unrealistic for the purposes of sleep monitoring as necessitated by the research [52,53], due to unfeasibility of wearing footwear while asleep. Wrist-worn placements were chosen for this study in keeping with the findings from a systematic review and practical considerations of device placement in [54], and the superiority for sleep quality-metrics from wrist-worn sensors as reported in [55]. Actiwatch 2 was selected for its reliability as a single device capable of 24 h activity and sleep monitoring and Verisense was chosen to investigate the potential match for that reliability while meeting the additional desirable functions for battery life, open-source algorithms and automatic data upload that is needed for further 24 h RPM in PA and sleep studies.

There are four aims of this study: (1) compare temporally matched PA measured via Verisense and Actiwatch 2 over the data collection period; (2) compare PA cut-points of sedentary, light and MVPA measured via Actiwatch 2 and Verisense over the data collection period; (3) evaluate the ability of Verisense to determine sleep time, wake time and total sleep time (TST) compared to sleep metrics measured by Actiwatch 2 and (4) evaluate the objective and subjective comparisons between sensor data and the participant diaries.

## 2. Materials and Methods

### 2.1. Participants

As previously reported in preliminary findings from Moore et al. [56], participants were recruited from the student population at Letterkenny Institute of Technology (LYIT) through word of mouth. Participants were required to satisfy the following inclusion criteria: (1) >18 years of age, (2) no self-reported condition(s) that could impede PA, and (3) no self-reported sleep issues. All participants were provided with information sheets that clearly defined the study protocols and objectives, and consent forms. Consenting participants were selected for inclusion for Free Living (FL) (n = 20) and Supervised Protocol (SP) (n = 16). There were zero participant withdrawals from the study. Five FL data sets were excluded from analysis due to a malfunction of one or both of the wearable sensors. Four incomplete SP data sets were excluded from analysis as a result of COVID-19 lockdown restrictions in Ireland impeding the completion of the study. A total of 15 valid sets of FL data and 12 valid sets of SP data were obtained from the participants for statistical analysis. Each participant was allocated a random participant ID. The study was approved by the Research Ethics Committee at LYIT. Descriptive variables include self-reported sex and age as collected by a study researcher before study commencement. Body mass index was calculated using measured height and weight of participants (kg/m^2^). 

### 2.2. Instrumentation

**Figure 1:**
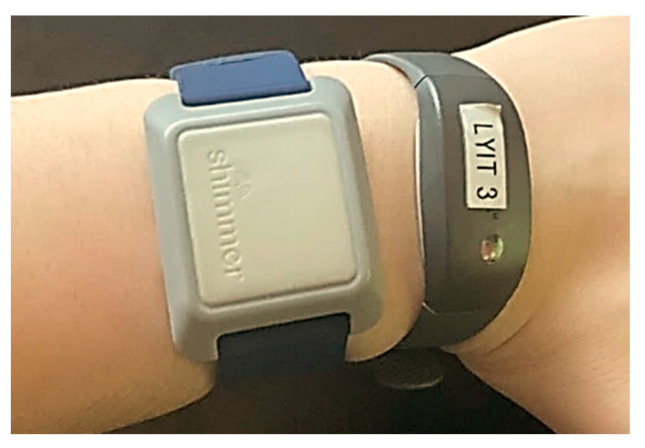
Verisense (left) and Actiwatch 2 (right) wrist-worn devices as worn by a participant.

An Actigraph is a portable device that records accelerometry data at the wrist. The Actiwatch 2 (Phillips Respironics Mini-Mitter) is a lightweight (16 grams) actigraphy device (43 mm × 23 mm × 10 mm as worn on wrist) that utilises a piezoelectric sensor to detect vertical accelerations spanning 0.5–2.0 g (Figure 1). The Actiwatch 2 has a rechargeable battery with a life of approximately 22 days at 15 s epochs and 30 days at 60 s epochs with a sampling rate of 32 Hz. Actiwatch 2 has a resistance rating of IPX7 meaning that it can be immersed in water for a maximum of 30 min between 15 cm to 1 m water depth [57]. Activity counts from the device are proprietary and reported peak acceleration is detected over each epoch to determine sleep and wake states [38]. Data are transferred from the devices to the Actiware software via USB docking station. 15 s epochs were used for data collection. The auto scoring setting on the Actiware software was used to determine sleep and wake from the Actiwatch 2 data and were used as the comparators. 

Verisense is a wrist-worn IMU which records PA and sleep duration (Figure 1). The Verisense system (Shimmer Research Ltd.) consists of a tri-axial accelerometer and gyroscope (29.6 g) (35 mm × 43 mm × 12 mm as worn on wrist) with an accompanying base station and accompanying software application that transfers data locally to Shimmers remote cloud system. The Verisense IMU sensor is a CE certified, Class 1 medical device [58] that records the wearer’s activity and sleep metrics onto an in-built flash memory, and once within range of the base station, data is automatically uploaded to the application on the base station. The base station then performs an automated data upload to an Amazon Web Services (AWS) server where data is then relayed to the relevant trial site and researchers have remote access to raw or analysed data for PA and sleep. Researchers can select the preferred sampling rate from the Verisense software with fixed values of 12.5 Hz, 25 Hz, 50 Hz, 100 Hz, 200 Hz, 400 Hz, 800 Hz and 1600 Hz. Verisense has a resistance rating of IP55 meaning that it is protected against water jets projected by a nozzle (6.3 mm) from any angle and is protected against dust that could interfere with the normal operation of the product but is not fully dust proof. The Verisense sensor has up to six months battery life with no need for recharging [59]. 

### 2.3. Study Protocol under Free-Living Conditions

As informed by previous research from [56] describing preliminary results, the validation study was divided into two testing sections: (1) a FL section and (2) a SP section. Each participant wore an Actiwatch 2 and Verisense sensors on the non-dominant wrist for the duration of both testing sections. Participants wore both sensors continuously for 48 h, resulting in two nights of sleep, and two days of PA data. For the study duration, 48 h was defined as the period spanning between midday on the first day until midday on the third day of measurement. Participants were requested to complete an accompanying PA and sleep diary during the FL section which was custom adapted by the research team based on commonly used sleep [60] and PA diaries [61,62] utilised in research. The diary included questions regarding time in bed, time lights out, time woke up, time lights on, time out of bed and total hours of sedentary, light and MVPA activity for each 24 h period. All participants adhered to this request. The outcome measures were (1) temporally aligned in 15 s epochs from both devices for the 48 h FL and SP, (2) aligned with PA levels for FL and SP using thresholds located in the literature, and (3) sleep and wake metrics from both devices using the participant diary as a guide [30] and using an open-source heuristic algorithm referred to as Heuristic algorithm looking at Distribution of Change in Z-Angle (HDCZA), without any accompanying sleep log to guide as described in detail elsewhere [63]. Participants were asked to remove the devices for any water-based activities and to record periods of non-wear in the adapted diary. Sleep and activity data were retrieved from the Verisense Dashboard [64] and the Actiwatch 2 data was obtained using Philips Actiware (v 6.0.9) [65]. Both devices were initialised prior to study commencement with internal clocks automatically synchronised to a research team computer. 

### 2.4. Study Protocol under Supervised-Protocol Conditions

Within the SP section, participants wore both the Actiwatch 2 and Verisense sensors on their non-dominant wrist for the duration of the gym-based activities shown in Table 1 and were supervised throughout by a study researcher. The walking and jogging activities were completed on treadmills and/or across a flat gym surface. Activities were completed in a randomised order as determined by study researchers. Participants were instructed by a study researcher when to start and stop each activity. The activity description, and exact start and finish times were recorded by the researcher. After each activity, participants were instructed to stand completely still with arms resting comfortably by the sides of the body for 60 s to accommodate a clear delineation between activity transitions in sensor outputs. The SP section was completed within a maximum of 45 mins, varying upon each participant’s walking/jogging pace for self-paced activities. The activities included were informed by previous studies which recommended various sedentary and locomotor activities listed in Table 1 [66]. Other activities suggested for inclusion in validation studies, such as bicycling were excluded from this study due to feasibility constraints, however ascending and descending of steps as recommended by [67] were included as feasibility permitted in the gym facilities utilised.

### 2.5. Data Reduction

Immediately following each participants test session, both sensors were removed from the participant, and the data were downloaded to a research team member’s computer using relevant software (Actiware software v6.5.2 and Verisense Cloud Platform). Actiwatch 2 activity counts were presented in timestamped .csv format in 15-second epochs. Raw triaxial acceleration values from Verisense were converted into one omnidirectional measure of body acceleration. For this, the vector magnitude (VM) was taken from the three axes and then subtracted by the value of gravity (g) as in Equation (1), √ (x^2^ + y^2^ + z^2^)-1g, after which, negative values were rounded up to zero, referred to as Euclidean norm minus one (ENMO). 

ENMO, described in detail elsewhere [27,68,69], where 1000 = 1000 milli-gravitational units = 1 gravitational unit, is defined as:

(1) $$r_{i}=\sqrt{x_{i}^{2}+y_{i}^{2}+z_{i}^{2}}=i^{th}$$

where: *i^th =^* vector magnitude at each time point.

For the computation of the ENMO metric, the resultant Verisense .csv files were combined into one .csv file using a custom script and values presented in m/s^2^ were converted to g using the same custom script, then exported into R statistical software V3.1.2 [70] for processing using the GGIR package (V2.2) [30,63,68,69,71,72]. The ENMO method subtracts a fixed offset value of 1 gravitational unit at each time point to correct for gravity [27,68,69]. Negative ENMO values are rounded up to zero to reduce bias and error [27,69]. By design, the ENMO metric is sensitive to poor calibration [69]. Therefore, to address these calibration issues, ENMO was calculated using the GGIR package, which auto-calibrates the raw triaxial accelerometer signal [69]. The package regenerated the time-stamps and the files were exported into SPSS Statistical software V26 [73] for analysis. Further information on the accelerometer calibration technique can be found elsewhere [69]. ENMO was expressed in mg and calculated over 15 s epochs as previously described in preliminary findings from Moore et al. [56] to facilitate epoch-by-epoch comparison between sensors.

### 2.6. Data Analysis

Spearman’s rank-order correlation coefficient [74,75] was used to assess the temporal relationship between activity counts from the Actiwatch 2, and ENMO from Verisense because the data were not normally distributed. In accordance with a well-documented scale for medical research, we considered correlations of 0–0.3 as negligible correlation, 0.3–0.5 as low correlation, 0.5–0.7 as moderate correlation, 0.7–0.9 as high correlation, and 0.9–1.0 as very high correlation. A *p* < 0.05 was considered statistically significant for all analyses [76]. 

PA classification were compared between devices using sensitivity, specificity and overall accuracy [77] as well as Spearman’s rank-order correlation coefficient. Sleep parameters were compared between each sensor with Spearman’s rank-order correlation coefficient. To evaluate the agreement between two measurement methods [76], Bland–Altman plots [78] were used to visually evaluate the agreement of the sleep summaries collected by the two sensors. This technique plots the difference score between two measures against their means. Sleep diary data were used to set the time in bed period. Actiwatch data were converted into “sleep” and “wake” using Actiware’s proprietary algorithms, previously explained in [38]. To determine the occurrence of wake and sleep states in Verisense, the open-source GGIR algorithm was used to automatically determine sleep onset time and sleep offset time, as widely validated in previous research both with and without corresponding sleep diaries [30,63,68,69,71,72]. 

## 3. Results

For the FL study, 15 participants were included (11 males, mean age (±SD) 23 (±3.4) years, mean BMI (±SD) 23.9 (±2.6) kg/m^2^, and 4 females, mean age (±SD) 29 (±12.6), mean BMI 22.6 (±1.3) kg/m^2^). For the SP study, 12 participants were included (11 males, mean age (±SD) 23 (±3.4) years, mean BMI (±SD) 23.9 (±2.6) kg/m^2^, and 1 female, mean age (±SD) 22 (±0), mean BMI 22.8 (±0) kg/m^2^). Everyone who participated in the SP study also participated in the FL study. All participants were third-level students. Activities performed by the participants during the test days included sitting (e.g., at lectures), standing (e.g., practical classes) and walking. A number of participants were highly active (e.g., did workouts) whereas others were mainly sedentary during the test days. 

**Figure 2:**
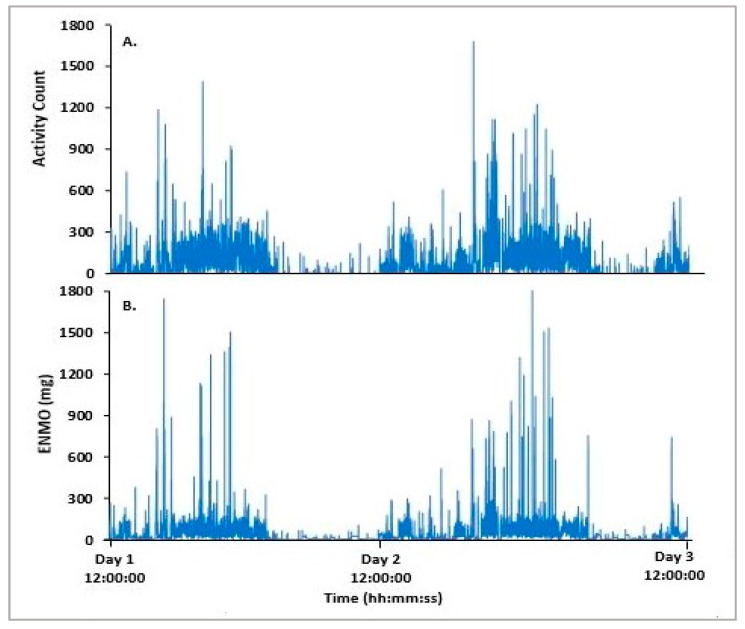
Representative epoch-by-epoch activity tracing of Actiwatch 2 (**A**) and Verisense (**B**) from a participant over a 48 h FL study.

**Figure 3:**
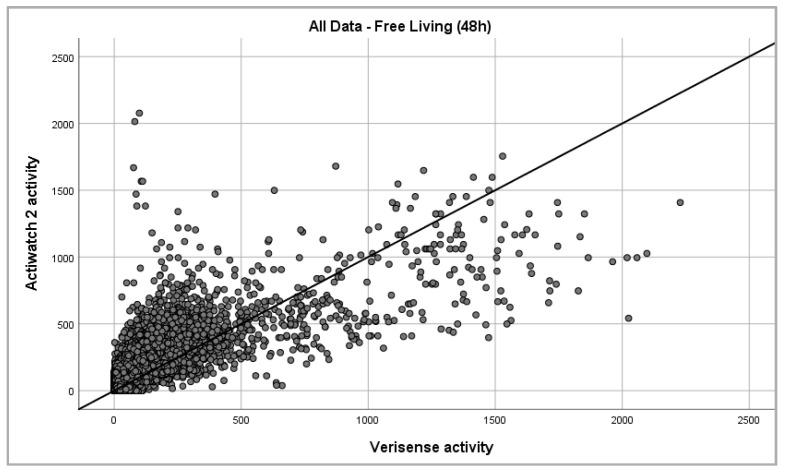
Epoch-by-epoch absolute activity of Actiwatch 2 and Verisense as recorded from all subjects over 48 h (163,759 data points).

The study compared epoch-by-epoch data obtained from both the Actiwatch 2 and Verisense devices over the 48 h FL study period from all 15 participants. The overall patterns observed between the Actiwatch 2 and Verisense visually appear to be quite similar for the 48 h FL study (Figure 2 and Figure 3). Movement data from 48 h FL absolute activity for the Actiwatch 2 and Verisense sensors were highly correlated (r = 0.85 ± 0.04, range: 0.77–0.92, n = 15; Spearman correlation). 

**Figure 4:**
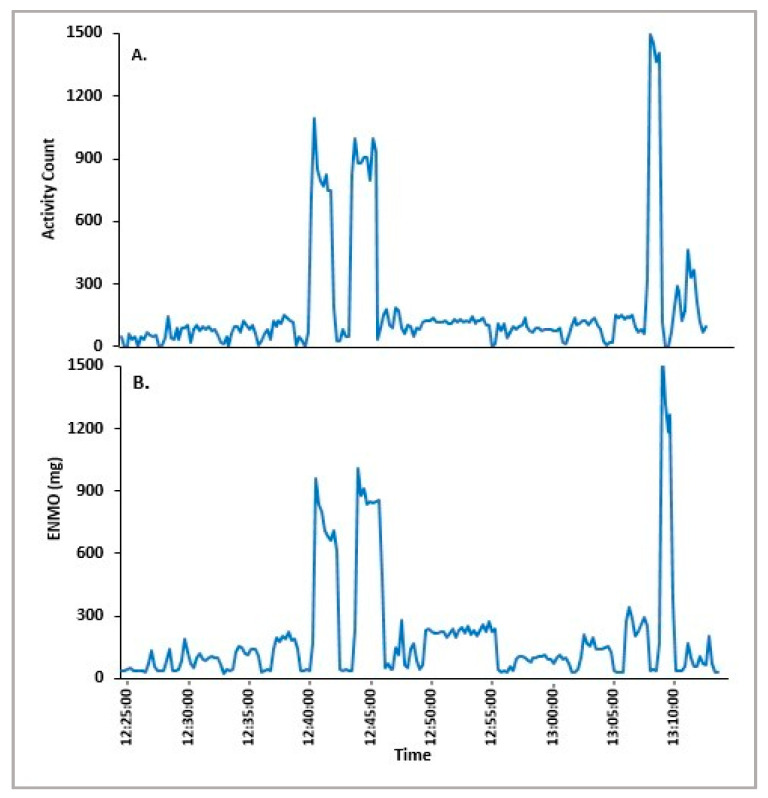
Representative epoch-by-epoch activity tracing of Actiwatch 2 (**A**) and Verisense (**B**) from a participant over a gym-based SP study.

**Figure 5:**
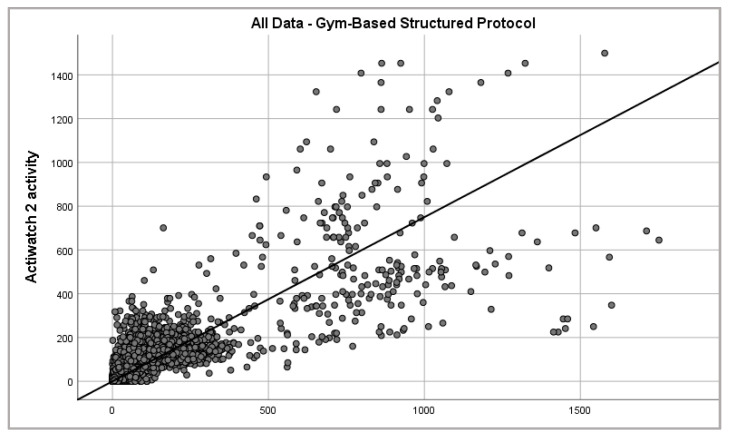
Epoch-by-epoch absolute activity of Actiwatch 2 and Verisense as recorded from all subjects over gym-based supervised protocol (2330 data points).

Epoch-by-epoch data obtained from both the Actiwatch 2 and Verisense sensors were compared over the gym-based SP from 12 participants. The overall patterns observed between the Actiwatch 2 and Verisense appear to be visually quite similar for the gym-based SP study (Figure 4 and Figure 5). Within participants, gym-based SP activity for the Actiwatch 2 and Verisense sensors were also highly correlated (r = 0.78 ± 0.05, range: 0.72–0.88, n = 12; Spearman correlation).

Epoch-by-epoch level data were segmented into PA levels of sedentary, light and MVPA activity using previously published cut-points from [36] for Actiwatch 2. These cut-points were defined as sedentary < 145, light <= 274, moderate > 274 and vigorous >= 597. However, limitations of sample size and female-only study participants is a notable limitation in the validity of application to these cut-points in other populations. For this reason, Actiwatch 2 cut-points could not be used as a gold-standard from which to base corresponding Verisense cut-points using Receiver Operating Characteristic (ROC) curves. All Verisense epoch-by-epoch level data were segmented using GGIR and processed in R following mean cut-points utilised by two similar specification accelerometers [27,28] of sedentary < 45, light <= 97, moderate > 97 and vigorous >= 423. To date, no cut-point validation has occurred for Verisense, hence these cut-points should be noted as experimental only. 

Using the aforementioned cut-point values, sensitivity, specificity and accuracy of the imputed PA levels were examined, as determined by the Actiwatch 2. For the most part, there was moderate correspondence in the determination of cut-points by Verisense and Actiwatch 2. The observed concordance between Verisense and Actiwatch 2 is presented in Table 2. 

Within FL participants, cut-point generated activity for the Actiwatch 2 and Verisense sensors were highly correlated for sedentary, low correlated for light and moderately correlated for MVPA. Within SP participants, cut-point generated activity for the Actiwatch 2 and Verisense sensors were low correlated for sedentary, negligibly correlated for light and low correlated for MVPA as presented in Table 2.

Epoch-by-epoch sleep metrics were calculated from the two sensors using both the sleep/wake classification proprietary algorithm for Actiwatch 2 and GGIR for Verisense. Using both algorithms, TST (min), sleep and wake times were reported from both sensors. TST are the epochs scored as sleep within the reported time span between sleep and wake. The agreement of the three sleep indicators (TST, sleep time and wake time) between participant diaries, Actiwatch 2, Verisense guided by participant diary and Verisense unguided was tested using Spearman correlation coefficients and Bland– Altman plots. To facilitate comparison, the sleep start time and wake time were converted from time to numerical values for statistical comparison. values were calculated for time duration from 18:00 until sleep start time, and time values from 00:00 until wake time. All times were converted to mins. No day sleepers were included in the study. 

Table 3, Table 4 and Table 5 shows the Spearman’s correlation coefficient of sleep start times, wake times and TST for participant diaries, Actiwatch 2, Verisense guided by participant diary and Verisense unguided. First, the total average sleep start times of all the participants were 402.5 ± 92.8 min, 417.4 ± 149.5 min, 390.7.5 ± 127.4 min and 398.9 ± 114.5 min, respectively. Next, the total average wake times of all the participants were 555.2 ± 96.7 min, 575.97 ± 115.8 min, 550.5 ± 118.4 min and 576.9 ± 118.8 min, respectively. Finally, the total average sleep times of all the participants were 515.1 ± 107.6 min, 524.8 ± 131.8 min, 526.6 ± 121.5 min and 543.1 ± 102.4 min, respectively.

**Figure 6:**
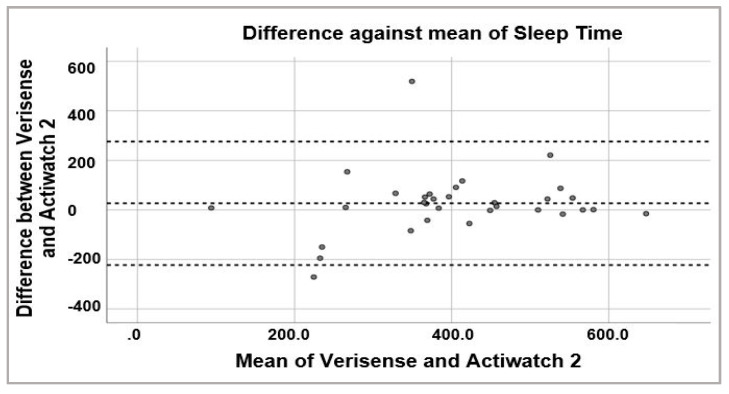
Bland–Altman plot of sleep times estimated by Verisense as compared to Actiwatch 2.

**Figure 7:**
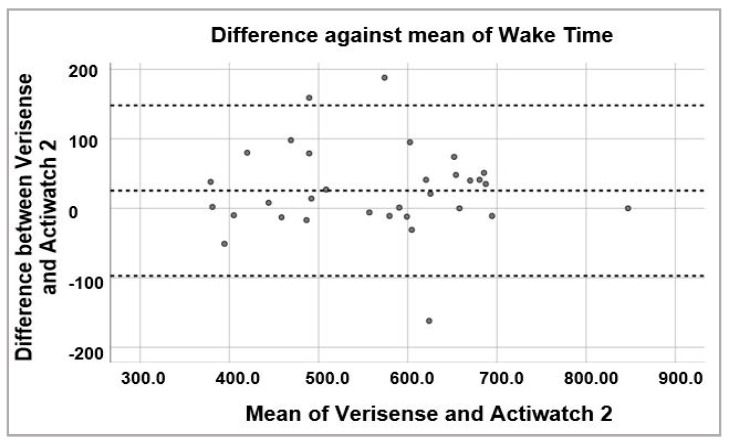
Bland–Altman plot of wake times estimated by Verisense as compared to Actiwatch 2.

**Figure 8:**
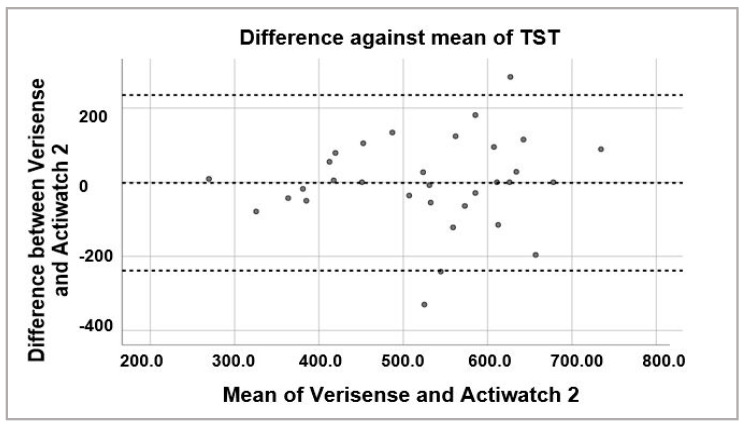
Bland–Altman plot of TST estimated by Verisense as compared to Actiwatch 2.

To examine the possibility of systematic bias in overall sleep parameter scoring, Bland–Altman plots were generated to visually inspect the level of agreement between Verisense and Actiwatch 2 results (Figure 6, Figure 7 and Figure 8). For sleep time, wake time and TST, the spread of the differences visually appears to be even, with no bias in overestimation or underestimation of sleep, wake or TST. 

## 4. Discussion

To our knowledge, this is the first validation study where the Verisense IMU was compared to an actigraph for activity and sleep monitoring. In comparing the accuracy of Verisense, a novel research-grade wearable sensor, against a clinical/research-grade actigraphy device, Actiwatch 2, we find that the former device performs similarly in the estimation of epoch-by-epoch activity scoring and sleep parameters, although future studies on PA level classifications need further examination. 

There are notable differences between the Verisense and the Actiwatch 2. While present on the Actiwatch 2, the Verisense lacks a light sensor, a feature often useful in identifying bed and wake times. The Actiwatch 2 stores data at a lower average resolution (e.g., 15 s and 30 s epochs at 32 Hz) in comparison to the Verisense which is capable of raw data monitoring and storage ranging up to 1600 Hz, facilitating higher resolution data with potential for greater accuracy. Verisense devices also remotely uploads all data to a secure cloud portal, eliminating the need for participants to attend a research facility to have data from the device downloaded, which is necessary with the Actiwatch 2. Significantly, Verisense provides access to raw accelerometer data which in place of a proprietary unit such as activity counts and filtered data which is now a desired part of research and increasing in adoption [45]. Verisense also has a long-lasting battery life of up to 6 months with no need for recharging, while most devices designed for clinical trials require frequent recharging and manual data upload. For longer duration longitudinal studies, these attributes of Verisense could be of significant benefit.

The adoption of wearable technology in healthcare and clinical trials continues to increase, however the paucity of standards for sensor algorithms can hamper their utilisation in research [79]. To address this, a healthcare industry open-wearables initiative (OWEAR) has been established [80]. The initiative seeks to develop open source algorithms and software for wearable sensor data analysis available to all medical device and pharmaceutical companies in a pre-competitive environment as a service to the industry [79,80,81]. 

One key area of debate in accelerometry activity monitoring is most appropriate wear-site for maximum accuracy [82,83]. Both devices presented in the study were wrist-worn, however many previous studies have utilised sensors worn at the waist [84,85]. It is therefore imperative to understand whether wrist-worn devices are an acceptable alternative compared to the waist for PA monitoring. In 2011, the U.S. National Health and Nutrition Examination Survey began using wrist-worn accelerometers to estimate PA [86]. Ref. [48] reported that a waist-worn GENEA triaxial accelerometer produced an almost identical correlation with energy expenditure as the same model worn on the wrist. However, Ref. [87] reported that a uniaxial accelerometer worn on the wrist and hip of participants during lifestyle activities produced a discrepancy in variance in energy expenditure with the waist-worn accelerometer accounting for 31.7% of the variance and the wrist-worn accelerometer explaining only 3.3% of the variance. This suggests that a triaxial accelerometer such as Verisense is suitable as a wrist-worn wearable device for PA. 

Future studies will compare Verisense to indirect caliometry for further PA classification validation, as this is the true, current gold standard in determination of PA cut-points [33,34,35]. The current results do, however, support the potential use of Verisense as an actigraphy device for the purpose of activity and sleep monitoring.

## 5. Conclusions

Verisense, a novel research-grade wearable device, produces activity and sleep parameters that are comparable to a research-grade actigraph and thus, can be used as a PA monitor and an actigraph for sleep monitoring.

It is a pertinent challenge to find a single reliable sensor to assess both PA and sleep while adhering to budgets and maximising participant compliance through minimising burden. We assert that this work is a step forward in examining and validating one single novel research-grade wearable device that provides access to raw sensor data, automated upload and long-lasting battery life which all contribute to negating participant burden, especially for longitudinal research. Concomitant validation of the Actiwatch 2 and Verisense against indirect caliometry will be an important future step to determine full equivalence of PA classification. 

## Figures and Tables

**Table 1:** Supervised protocol activities.

Activity	Time (min)
Sit	2
3.5 km/h walk on treadmill	2
4.5 km/h walk on treadmill	2
5.5 km/h walk on treadmill	2
7.5 km/h walk on treadmill	2
11.5 km/h walk on treadmill	2
Walking on flat surface at regular pace	6
200 m slow walk on flat surface	
200 m normal walk on flat surface	
200 m fast walk on flat surface	
200 m jog walk on flat surface	
Ascend 15 steps	
Descend 15 steps	

**Table 2:** Overall accuracy, sensitivity and specificity comparative performance and Spearman’s correlation coefficient and range values of Verisense in detecting PA levels during FL and SP, in comparison to Actiwatch 2.

		Overall Accuracy % *	Sensitivity % *	Specificity % *	Spearman’s *	Range *
**Sedentary**	**FL**	92.9	93.2	90.9	0.72 ± 0.05	0.65–0.83
	**SP**	56.0	35.6	96.4	0.36 ± 0.016	0.11–0.63
**Light**	**FL**	90.4	51.3	93.9	0.42 ± 0.06	0.27–0.53
	**SP**	65.2	14.4	79.9	−0.04 ± 0.10	−0.27–0.12
**MVPA**	**FL**	95.8	84.8	96.0	0.52 ± 0.09	0.35–0.78
	**SP**	65.0	95.2	63.1	0.49 ± 0.13	0.16–0.60

* Accuracy, sensitivity, specificity and Spearman’s of Verisense as compared to Actiwatch 2

**Table 3:** Sleep correlations.

Sleep Time	Participant Diary	Actiwatch 2	Verisense—Guided	Verisense—Unguided
**Participant Diary**	-	0.53	0.79	0.46
**Actiwatch 2**	0.53	-	0.66	0.43
**Verisense—Guided**	0.79	0.66	-	0.67
**Verisense—Unguided**	0.46	0.43	0.67	-

*p* < 0.01, correlation coefficient (r = 0.00–0.30: negligible correlation, r = 0.30–0.50: low correlation, r = 0.50–0.70: moderate correlation, r = 0.70–0.90: high correlation, r = 0.90–1.00: very high correlation)

**Table 4:** Wake correlations.

Wake Time	Participant Diary	Actiwatch 2	Verisense—Guided	Verisense—Unguided
**Participant Diary**	-	0.89	0.90	0.80
**Actiwatch 2**	0.89	-	0.83	0.80
**Verisense—Guided**	0.90	0.83	-	0.82
**Verisense—Unguided**	0.80	0.80	0.82	-

*p* < 0.01, correlation coefficient (r = 0.00–0.30: negligible correlation, r = 0.30–0.50: low correlation, r = 0.50–0.70: moderate correlation, r = 0.70–0.90: high correlation, r = 0.90–1.00: very high correlation).

**Table 5:** TST correlations.

Total Sleep Time	Participant Diary	Actiwatch 2	Verisense—Guided	Verisense—Unguided
**Participant Diary**	-	0.46	0.75	0.44
**Actiwatch 2**	0.46	-	0.54	0.31
**Verisense—Guided**	0.75	0.54	-	0.63
**Verisense—Unguided**	0.44	0.31	0.63	-

*p* < 0.01, correlation coefficient (r = 0.00–0.30: negligible correlation, r = 0.30–0.50: low correlation, r = 0.50–0.70: moderate correlation, r = 0.70–0.90: high correlation, r = 0.90–1.00: very high correlation).

## Data Availability

The datasets generated during and/or analysed during the current study are available from the corresponding author on reasonable request.

## Abbreviations

The following abbreviations are used in this manuscript:

ENMO	Euclidean Norm Minus One
FL	Free-living
SP	Supervised Protocol
TST	Total Sleep Time
IMU	Inertial Measurement Unit
SB	Sedentary Behaviour
PA	Physical Activity
PSG	Polysomnography
